# Activation mechanism dependent surface exposure of cellular factor XIII on activated platelets and platelet microparticles

**DOI:** 10.1111/jth.15668

**Published:** 2022-02-21

**Authors:** Laura Somodi, Ildikó Beke Debreceni, Gréta Kis, Marco Cozzolino, János Kappelmayer, Miklós Antal, György Panyi, Helga Bárdos, Nicola J. Mutch, László Muszbek

**Affiliations:** ^1^ 443158 Division of Clinical Laboratory Science Faculty of Medicine University of Debrecen Debrecen Hungary; ^2^ 443158 Department of Laboratory Medicine Faculty of Medicine University of Debrecen Debrecen Hungary; ^3^ 443158 Kálmán Laki Doctoral School of Biomedical and Clinical Sciences University of Debrecen Debrecen Hungary; ^4^ 443158 Department of Anatomy, Histology and Embryology Faculty of Medicine University of Debrecen Debrecen Hungary; ^5^ 443158 Department of Biophysics and Cell Biology Faculty of Medicine University of Debrecen Debrecen Hungary; ^6^ 443158 Department of Public Health and Epidemiology Faculty of Medicine University of Debrecen Debrecen Hungary; ^7^ Aberdeen Cardiovascular and Diabetes Centre School of Medicine Medical Science and Nutrition Institute of Medical Sciences University of Aberdeen Aberdeen UK

**Keywords:** cell‐derived microparticles, factor XIII, flow cytometry, immune electron microscopy, platelet activation

## Abstract

**Background:**

Platelets contain a high amount of potentially active A subunit dimer of coagulation factor XIII (cellular FXIII; cFXIII). It is of cytoplasmic localization, not secreted, but becomes translocated to the surface of platelets activated by convulxin and thrombin (CVX+Thr).

**Objective:**

To explore the difference in cFXIII translocation between receptor mediated and non‐receptor mediated platelet activation and if translocation can also be detected on platelet‐derived microparticles. Our aim was also to shed some light on the mechanism of cFXIII translocation.

**Methods:**

Gel‐filtered platelets were activated by CVX+Thr or Ca^2+^‐ionophore (calcimycin). The translocation of cFXIII and phosphatidylserine (PS) to the surface of activated platelets and platelet‐derived microparticles was investigated by flow cytometry, immunofluorescence, and immune electron microscopy. Fluo‐4‐AM fluorescence was used for the measurement of intracellular Ca^2+^ concentration.

**Results:**

Receptor mediated activation by CVX+Thr exposed cFXIII to the surface of more than 60% of platelets. Electron microscopy revealed microparticles with preserved membrane structure and microparticles devoid of labeling for membrane glycoprotein CD41a. cFXIII was observed on both types of microparticles but was more abundant in the absence of CD41a. Rhosin, a RhoA inhibitor, significantly decreased cFXIII translocation. Non‐receptor mediated activation of platelets by calcimycin elevated intracellular Ca^2+^ concentration, induced the translocation of PS to the surface of platelets and microparticles, but failed to expose cFXIII.

**Conclusions:**

The elevation of intracellular Ca^2+^ concentration is sufficient for the translocation of PS from the internal layer of the membrane, while the translocation of cFXIII from the platelet cytoplasm requires additional receptor mediated mechanism(s).

**Essentials**

- The cellular form of factor XIII (cFXIII) is of cytoplasmic localization in resting platelets.
- Surface exposure of cFXIII was revealed by flow cytometry, immunofluorescence, and immune electron microscopy.
- Activation by convulxin+thrombin but not by Ca^2+^‐ionophore exposes cFXIII to the surface of platelets and microparticles.
- Biochemical pathways preceding intracellular Ca^2+^ release are required for cFXIII translocation.

## 1 INTRODUCTION

Coagulation factor XIII (FXIII) exists in two forms.^1^, ^2^, ^3^, ^4^ The plasmatic form is a tetramer that consists of two potentially active A subunits and two protective/inhibitory B subunits (FXIII‐A_2_B_2_). Proteolytic removal of the activation peptide from the N‐terminus of FXIII‐A by thrombin and the dissociation of the heterotetramer in the presence of Ca^2+^ transforms plasma FXIII into a transglutaminase that by cross‐linking fibrin chains and α_2_‐antiplasmin to fibrin plays an essential role in fibrin stabilization. The existence of a cellular form of FXIII (cFXIII) in platelets has been known for some time^5^, ^6^ and it has since been described in other cell types including monocytes, macrophages, osteoblasts, osteocytes, chondrocytes, preadipocytes, and corneal keratocytes.^4^, ^7^, ^8^, ^9^, ^10^, ^11^, ^12^, ^13^ cFXIII is a dimer of FXIII‐A, the activation of which does not need proteolytic intervention—the elevation of intracellular Ca^2+^ concentration is sufficient to transform it into an active transglutaminase.^14^, ^15^

Platelets represent a huge reservoir of cFXIII, 60 fg FXIII‐A/individual platelet corresponds to 3% of the total platelet protein.^16^ In non‐activated resting platelets cFXIII is of cytoplasmic localization, while activation by strong stimuli, like convulxin (CVX) plus thrombin (Thr) results in its translocation to the membrane and partial externalization to the outer membrane layer.^17^ Using α_2_‐antiplasmin or an α_2_‐antiplasmin derived peptide it was also shown that surface‐exposed cFXIII represented active transglutaminase and could be involved in thrombus stabilization.^17^, ^18^ These two agonists induce platelet activation via binding to their receptors. CVX stimulates the collagen receptor glycoprotein VI (GPVI)/FcRγ generating a downstream signal igniting platelet activation process while Thr stimulation involves the protease‐activated receptors PAR1, PAR4, and GPIb.^19^ Stimulation of these receptors by CVX+Thr represents a robust signal leading to the activation of a number of signaling pathways involving protein phosphorylation and a strong elevation of intracellular Ca^2+^ concentration due to its release from intracellular pools. These signaling events result in aggregation of platelets, secretion of their granular content, the transposition of procoagulant phosphatidylserine (PS) to the outer membrane leaflet, and microparticle production. Influx of extracellular Ca^2+^ also contributes to these events.^20^, ^21^ Platelet activation can also be carried out by non‐receptor mediated mechanisms. The Ca^2+^‐ionophore calcimycin (A23187) is a receptor‐independent activation agent that elevates Ca^2+^ concentration in the platelet cytoplasm and ignites Ca^2+^‐dependent biochemical pathways of the activation machinery. Here we used flow cytometry, immunofluorescence, and immune electron microscopic techniques to study the externalization of cFXIII on activated platelets and on microparticles formed during platelet activation. Differences between the effects and the mechanisms of receptor mediated and non‐receptor mediated platelet activations were also explored.

## 2 METHODS

### 2.1 Materials

Sepharose CL‐2B column, bovine thrombin, and Ca^2+^‐ionophore calcimycin were from Sigma‐Aldrich. CVX was the product of Pentapharm. Phycoerythrin (PE)‐labeled anti‐CD62 antibody (Ab), PE‐cyanine5 (PECy5)‐conjugated anti‐CD41a Ab, PE‐labeled mouse IgG_1_, fluorescein isothiocyanate (FITC)‐labeled mouse IgG_2a_, PE‐conjugated annexin V, and annexin V binding buffer were purchased from Becton Dickinson. Mouse anti‐human CD41a Ab, Alexa‐fluor 568 annexin V conjugate, and DyLight 405‐labeled goat anti‐mouse Ab were the products of Thermo Fisher Scientific. DyLight 488‐labeled horse anti‐rabbit Ab, Vectashield^®^ antifade mounting medium, and normal goat serum were from Vector Laboratories. Goat anti‐rabbit IgG conjugated to 15 nm gold particles and goat anti‐mouse IgG conjugated to 10 nm gold particles were purchased from BBI Solutions. Uranyl acetate was from Electron Microscopy Sciences. Lead citrate was made from lead nitrate (VWR International Ltd.). Rabbit anti‐human FXIII‐A Ab and FITC‐labeled mouse anti‐human‐FXIII‐A Ab were produced in our laboratories.^16^, ^22^ The fluorescent calcium indicator Fluo‐4‐AM was obtained from Thermo Fisher Scientific. The RhoA inhibitor Rhosin hydrochloride was purchased from Tocris Bioscience and the transglutaminase inhibitor T101 from Zedira.

### 2.2 Preparation and activation of gel‐filtered platelets

Blood samples were drawn from healthy volunteers who had not taken any antiplatelet medications in the previous 14 days. Blood sampling was approved by the Ethics Review Board of the University of Debrecen, Faculty of Medicine in accordance with the Declaration of Helsinki. Peripheral blood was collected into vacutainer tubes containing 0.109 M sodium citrate anticoagulant (Vacutainer^®^; Becton Dickinson). After 1:1 dilution with HEPES buffer (10 mM HEPES, 136 mM NaCl, 2.7 mM KCl, 2 mM MgCl_2_, 0.1% glucose, pH 7.45) platelet‐rich plasma (PRP) was separated by centrifugation at 170 *g* for 10 min at 25°C. Platelets were isolated from PRP by gel filtration on Sepharose CL‐2B column equilibrated with HEPES buffer. Then, gel‐filtered platelets (GFPs) were incubated at 37°C for 30 min to recover their original shape.

GFPs in HEPES buffer supplemented with 2 mM CaCl_2_ and 0.1% bovine serum albumin (BSA) were stimulated by 125 ng/ml CVX, 0.5 U/ml thrombin, or their combination for 15, 30, or 45 min at 37°C. In other experiments platelets were activated by various concentrations of calcimycin. In certain experiments, immediately prior to activation by CVX+Thr, platelets were pretreated by Rhosin or T101. The results were compared to those obtained with non‐pretreated platelets.

### 2.3 Flow cytometric analysis

P‐selectin expression on activated platelets and platelet‐derived microparticles (MPs) was detected by co‐staining with PE‐labeled anti‐CD62 Ab (1:35 dilution) and by the platelet marker anti‐CD41a Ab conjugated to PECy5 (1:35 dilution). PE‐labeled mouse IgG_1_ (1:35 dilution) was used as isotype control. Staining for FXIII‐A, for CD41a, and PS detection by annexin V binding were performed in a triple immunofluorescent labeling system, using FITC‐labeled anti‐human‐FXIII‐A Ab (2 μg/ml), PE‐conjugated annexin V (1:35), and PECy5‐conjugated anti‐CD41a Ab. In this case FITC‐labeled mouse IgG_2a_ (1:35) served as isotype control. The antibodies were present during the respective platelet activation after which samples were diluted 12‐fold with annexin V binding buffer (10 mM HEPES, 140 mM NaCl, 2.5 mM CaCl_2_, pH 7.4, Becton Dickinson) and analyzed on a Beckman Coulter FC500 flow cytometer equipped with CXP Analysis software (Beckman Coulter). Platelets were gated based on their labeling for CD41a, aggregates were excluded on the basis of characteristic forward versus side scatter pattern. Platelet‐derived MPs were identified as platelet‐specific marker (CD41a) positive events with the size below 1 μm. Reference beads of 0.6, 1.1, and 3 μm (Sigma‐Aldrich) were used for calibration. Results were expressed as mean percentage of FXIII‐A positive platelets or MPs. Statistical analysis was carried out by GraphPad Prism 8.01. Distribution of the results was investigated by the Kolmogorov‐Smirnov test; non‐paired *t*‐test or Mann‐Whitney test was used for calculating the level of significance.

### 2.4 Immunofluorescent analysis

GFPs in HEPES buffer (with 2 mM CaCl_2_ and 0.1% BSA) were incubated at 37°C for 15 min in the absence or presence of 125 ng/ml CVX plus 0.5 U/ml thrombin or 0.7 μM calcimycin. Samples were diluted 10‐fold with HEPES buffer to stop cell activation. The cells and the formed MPs were stained with rabbit anti‐human FXIII‐A antibody (1:200 dilution), mouse anti‐human CD41a antibody (1:25 dilution), and Alexa‐fluor 568 annexin V conjugate (1:20 dilution) for 60 min. Then, platelets and vesicles were immobilized onto glass slides (4 × 10^4^ cells/slide) by Cytospin 3 cytocentrifuge (Shandon) at 72 × *g* for 4 min and slides were air‐dried. Immune labeling was visualized by incubation with DyLight 405‐labeled goat anti‐mouse and DyLight 488‐labeled horse anti‐rabbit secondary antibodies (both in 1:100 dilution) for 45 min. Subsequently, slides were washed and covered with Vectashield^®^ antifade mounting medium (Vector Laboratories). Phosphate‐buffered saline (PBS) was used for washing and for the dilution of antibodies. Staining steps were performed at room temperature in the dark. Images were taken by Zeiss LSM 700 confocal microscope (Zeiss) through Plan‐Apochromat 63x/1.40 oil immersion objective and analyzed by Zen software.

### 2.5 Double immunogold staining for electron microscopy

GFPs were processed for electron microscopic investigation as described for immunofluorescent analysis. Equal volumes of activated and non‐activated GFPs and 8% paraformaldehyde solution dissolved in 0.01 M PBS were carefully mixed and stored overnight at 4°C. Five microliters of the mixture was placed onto a carbon coated nickel 200 mesh grid (Electron Microscopy Sciences). After waiting 10 min for the deposition of platelets and extracellular vesicles onto the carbon layer, the rest of the liquid was removed by blotting. The grids were washed and incubated in a blocking solution containing 1% ovalbumin in 0.01 M PBS for 30 min. Platelets and extracellular vesicles on the grids were reacted with a mixture of rabbit anti‐FXIII‐A (1:5 dilution) and mouse anti‐CD41a (1:20 dilution) for 90 min. After several washes, the samples were incubated with a mixture of goat anti‐rabbit IgG conjugated to 15 nm gold particles (1:20 dilution) and goat anti‐mouse IgG conjugated to 10 nm gold particles (1:20 dilution) for 120 min. Both the primary and secondary antibodies were dissolved in 0.01 M PBS containing 1% normal goat serum and the incubations were performed at room temperature. After several washes, the samples were counterstained with uranyl acetate for 30 min and lead citrate for 5 min, then they were investigated in a JEOL 1010 transmission electron microscope (JEOL) and photographed with an in‐column Olympus Valeta CCD camera.

### 2.6 Intracellular Ca^2+^ measurement

The platelet count was adjusted to 30,000/μL with HEPES‐Ca^2+^ buffer (2 mM CaCl_2_) in a polypropylene tube and Fluo‐4‐AM was added to a final concentration of 3 mM. The cells were gently mixed and incubated for 15 min at room temperature in the dark. Afterward, PECy5 anti‐CD41a (final dilution 1:20) was added and the cells were incubated for an additional 15 min. The platelet count was then adjusted to 6.000/μL with HEPES‐Ca^2+^ buffer and acquired for 60 s on a Novocyte 3000 RYB flow cytometer (Agilent Technologies; λ_excitation_ = 488 nm; fluorescence channels: λ_emission_ = 530/30 for Fluo‐4 and λ_emission_ = 660/20 for Cy5). Acquisition was interrupted when activating the platelets with CVX+Thr (125 ng/ml and 0.5 U/ml, respectively) or after adding calcimycin (0.7 µM) and continued for at least 7.5 min. The technical time gap between the baseline and the acquisition following activation was 91 s. The results were analyzed by FlowJo software (Becton Dickinson). The gating strategy for CD41a‐positive platelets is illustrated in Figure S3 in supporting information.

## 3 RESULTS

### 3.1 Surface exposure of activation markers following platelet stimulation

**FIGURE 1:**
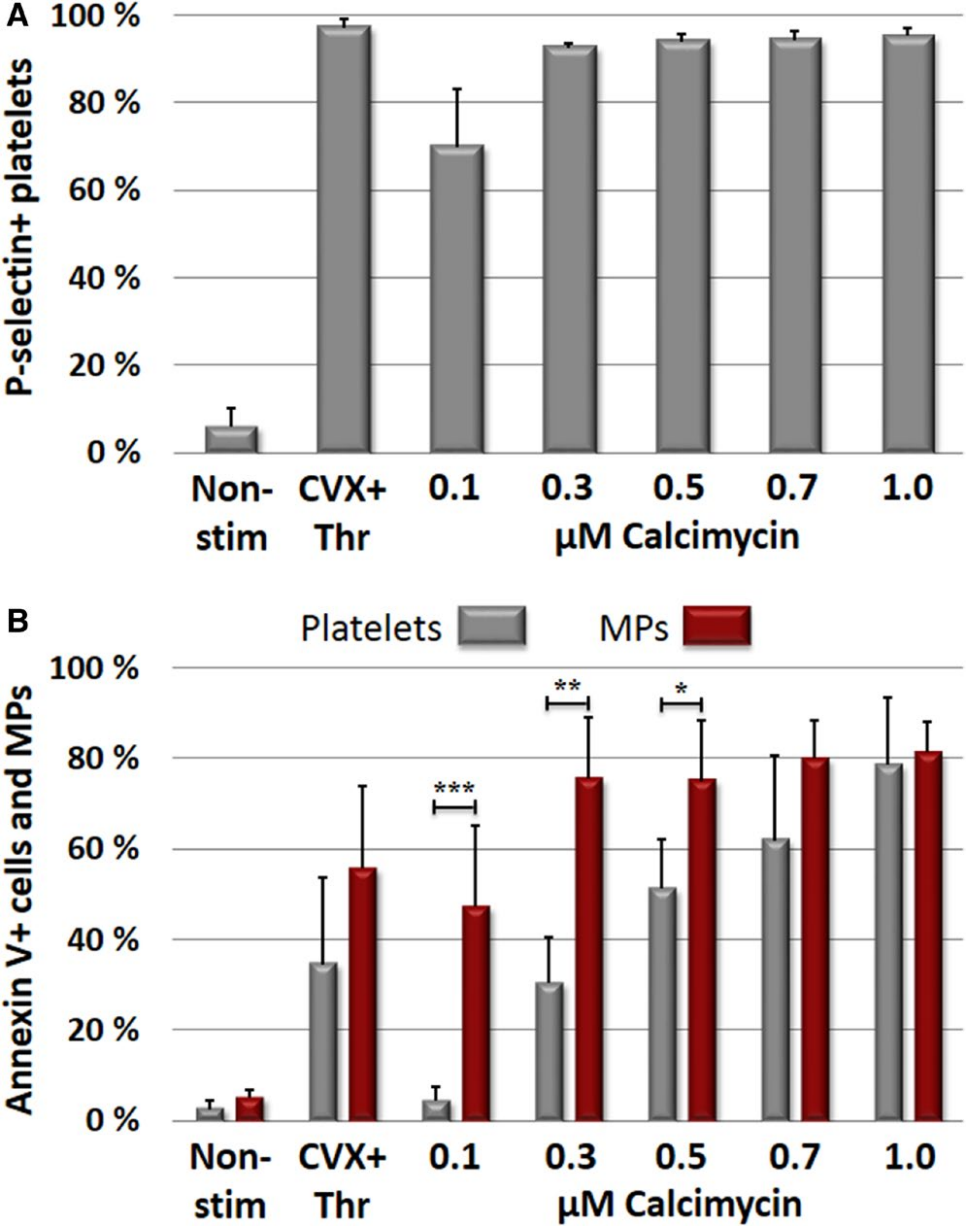
Receptor and non‐receptor mediated exposure of activation markers to the platelet surface. Gel‐filtered platelets were activated by convulxin+thrombin (CVX+Thr; receptor mediated activation) or by various concentrations of calcimycin (non‐receptor mediated activation) and surface exposure of activation markers was detected by flow cytometry. Results with non‐stimulated platelets (Non‐stim) were also shown. A, P‐selectin availability on the surface of activated platelets. B, Exposure of phosphatidylserine on activated platelets and platelet microparticles (MPs) as detected by annexin V binding. The columns and error bars represent means ± standard deviations calculated from the results of five different donors. Significant differences between platelets and microparticles are indicated on the figure *P < .05, **P < .01, ***P < .001

Flow cytometry revealed that both receptor mediated platelet activation induced by the CVX+Thr and non‐receptor mediated platelet activation induced by Ca^2+^‐ionophore resulted in the surface exposure of the α–granular protein P‐selectin (Figure 1A); 0.3 µM calcimycin was sufficient for exposing P‐selectin to the surface of >90% of platelets, comparable to stimulation with CVX+Thr. In contrast, a higher calcimycin concentration (0.7 µM) was required to induce close to maximal platelet aggregation (Figure S1 in supporting information). Exposure of the anionic phospholipid PS on the surface of activated platelets was quantified by binding of annexin V. As demonstrated by this technique, such transposition was detected in one third of CVX+Thr activated platelets and in 61% of the platelets activated by 0.7 µM calcimycin (Figure 1B). At lower calcimycin concentration the percentage of PS‐positive microparticles was significantly higher than PS‐positive platelets.

### 3.2 Receptor‐mediated transposition of cFXIII to the surface of activated platelets and platelet microparticles

**FIGURE 2:**
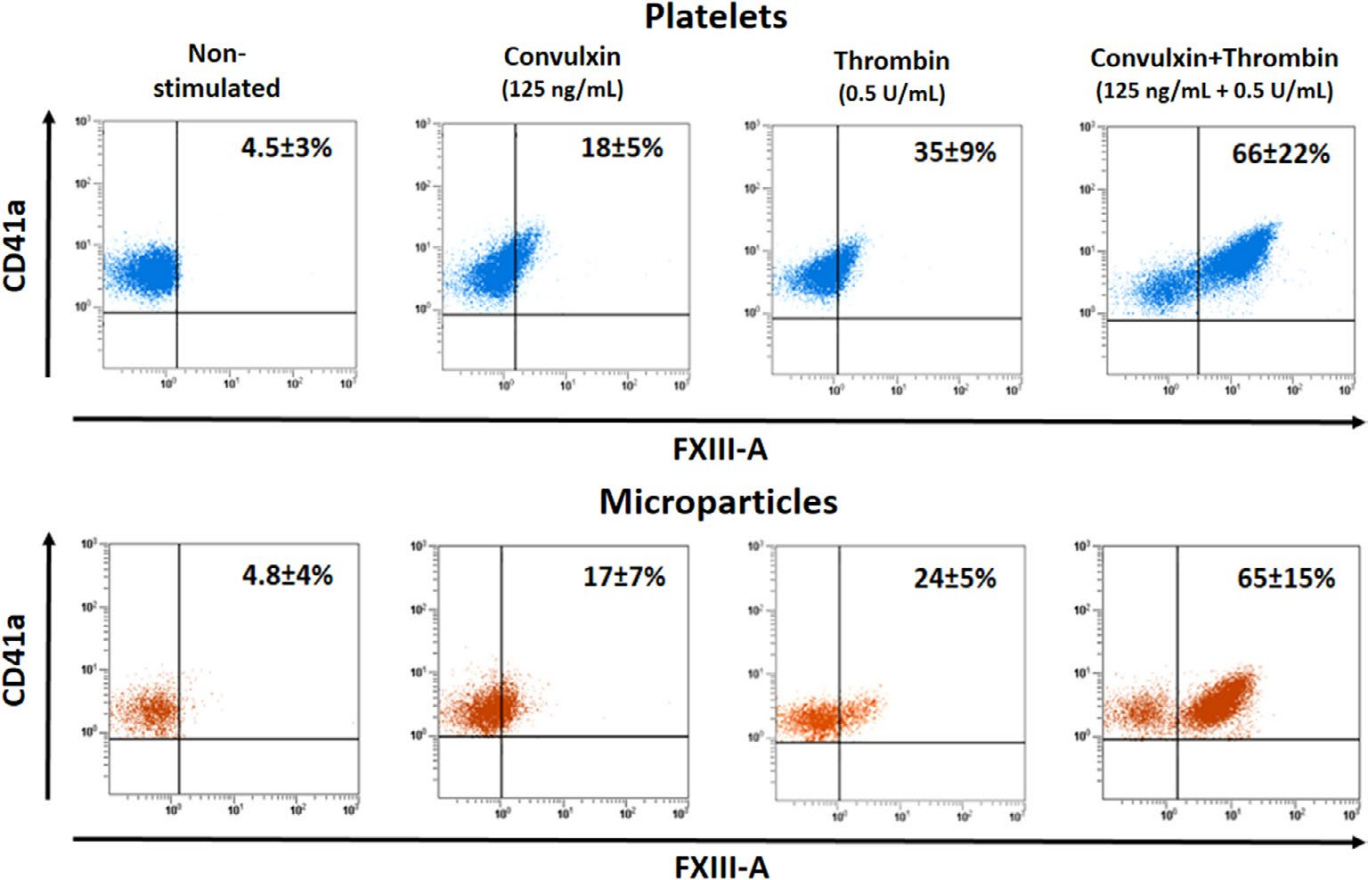
Surface exposure of factor XIII A subunit (FXIII‐A) on platelets activated by receptor mediated mechanism and on the formed microparticles. In each case platelets were activated for 15 min. The detection of CD41a antigen was used for the identification of platelets and platelet‐derived microparticles. Microparticles were gated at 1 µm. The upper row demonstrates the results with activated platelets, while in the lower row results obtained with the formed microparticles are shown. The dot plots show the results of representative individual samples, while the numbers in the right upper corners represent the means ± standard deviations; each value was calculated from measurements on samples from five different donors. With the exception of thrombin activation there was no significant difference between FXIII‐A positivity of platelets and microparticles. In the case of thrombin induced activation, the difference of platelets versus microparticles was significant (*P* = .038)

Flow cytometric analysis of platelets and platelet microparticles for surface exposed cFXIII revealed that both CVX and Thr alone were able to induce transposition of cFXIII in a significant percentage of activated platelets and microparticles (Figure 2). The combined results obtained with samples from five different donors are numerically indicated on the dot plots. However, the dual stimulus through the GPVI/FcRγ and PAR1 plus PAR4 receptors was needed to elicit a robust effect with >60% of platelets and platelet microparticles exposing cFXIII. It seems that only the combination of CVX+Thr was able to produce a distinct platelet population that emerges as the combination of two separate platelet populations representing functionally different entities. Such surface distribution is similar to what was observed with alpha granular factor V.^23^

**FIGURE 3:**
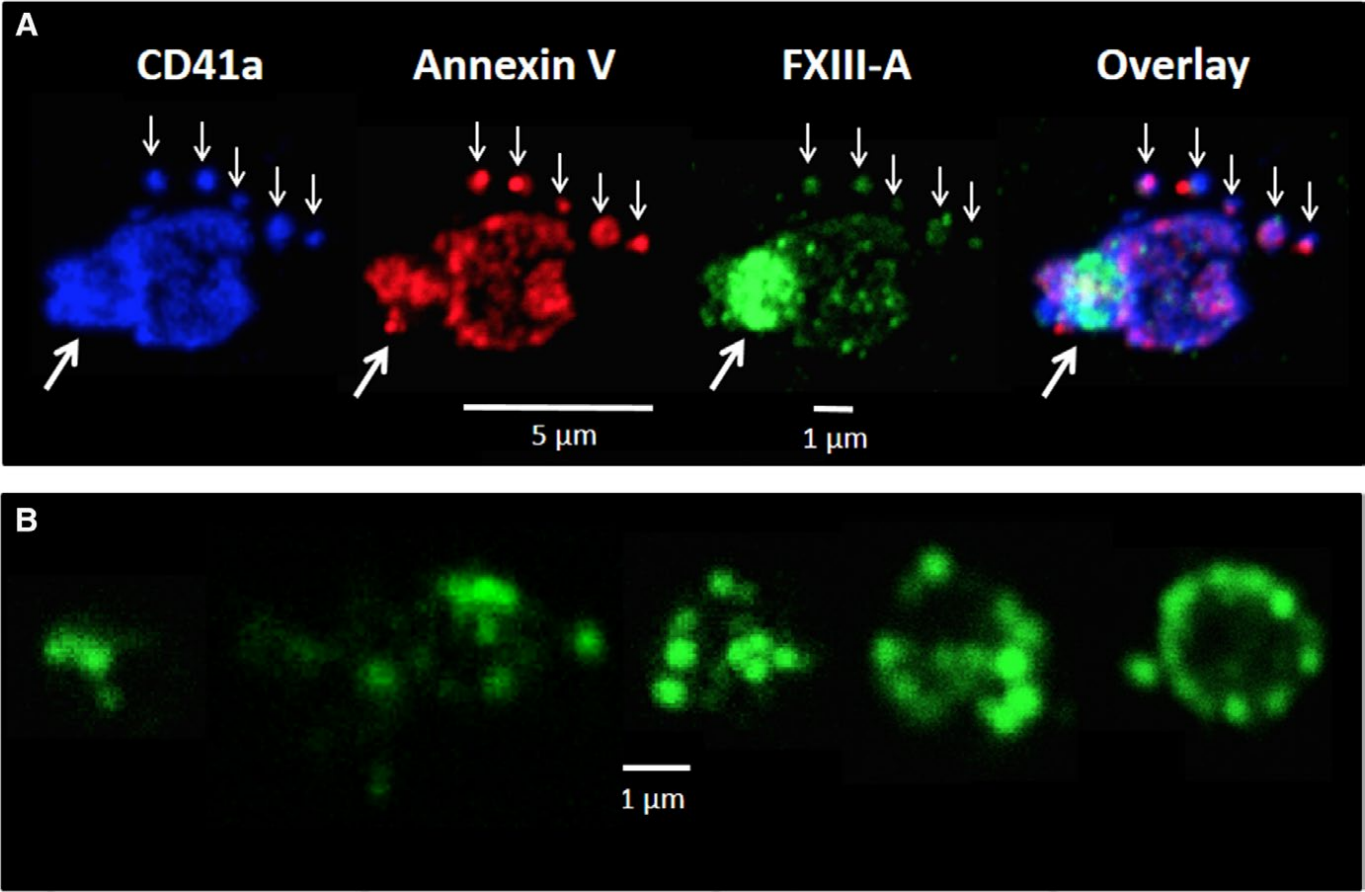
Surface labeling of platelets activated by receptor mediated mechanism and of the formed microparticles using immunofluorescent markers. A, Platelet activated by convulxin+thrombin stained for CD41a (blue), annexin V binding (phosphatidylserine labeling, red) and factor XIII A subunit (FXIII‐A; green). The small white arrows point to microparticles, while the thick white arrows point to the cap‐like structure on an activated platelet. B, FXIII‐A positive clumped microparticles formed from platelets activated by convulxin+thrombin. The images are representative of experiments performed on platelets from three different donors

Triple immunofluorescent staining for CD41a, annexin V, and cFXIII on CVX+Thr stimulated platelets is shown in Figure 3A. A strong signal for PS over the surface of the membrane is consistent with procoagulant platelet formation. CD41a stained all over the surface of the procoagulant platelet, while cFXIII was concentrated within the cap‐like structure protruding from the cell (Figure 3A). CD41a‐ and PS‐positive microparticles of approximately 700–900 nm could also be observed in Figure 3A. Consistent with the flow cytometry data these microparticles also showed surface staining for FXIII‐A. The resolution of immunofluorescence is not sufficient to explore microparticles of smaller size, but on the slides prepared by cytocentrifuge we were able to detect clumped microparticles of up to 2 µm diameter, which showed intensive staining for FXIII‐A (Figure 3B).

**FIGURE 4:**
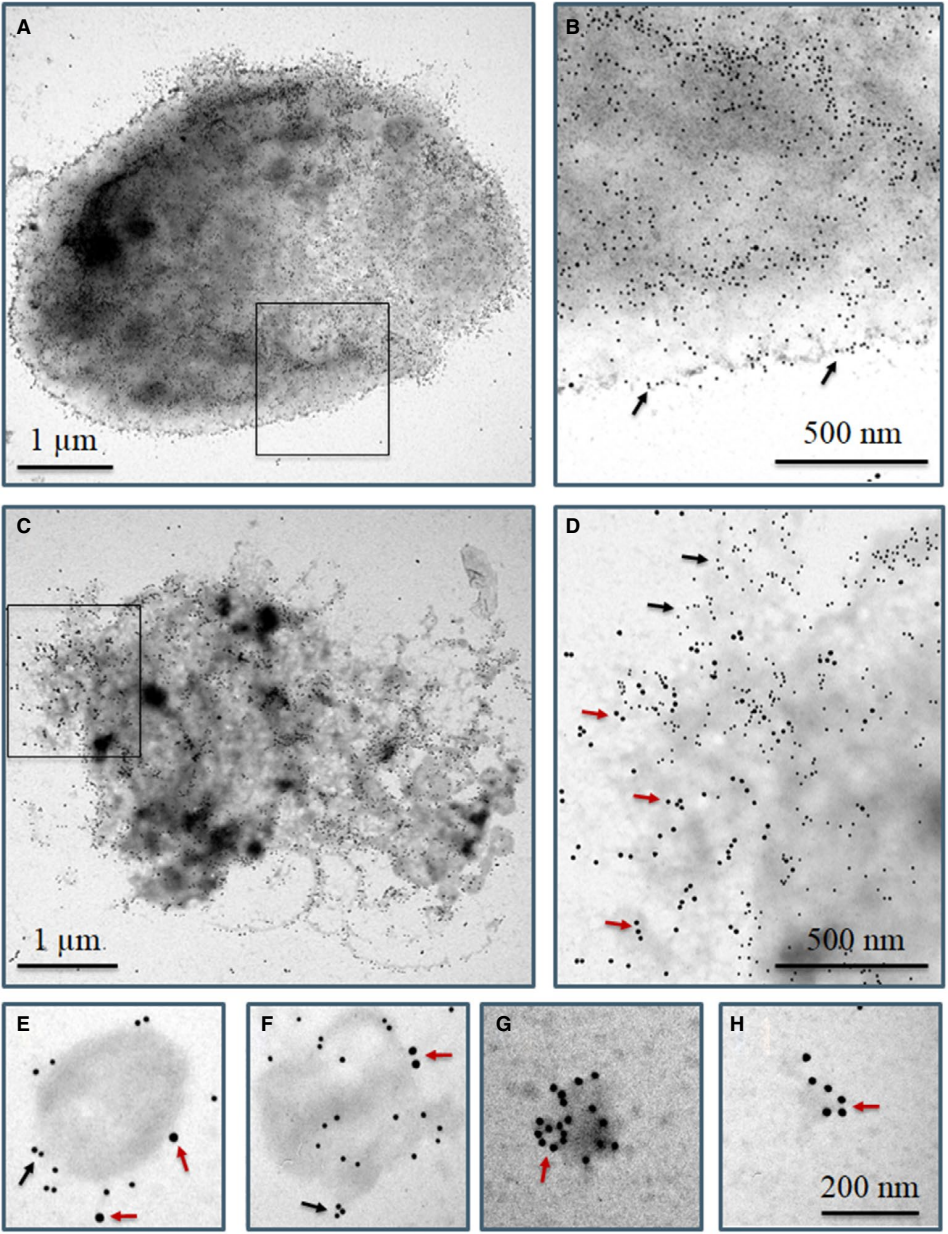
Surface labeling of resting and convulxin+thrombin (CVX+Thr) activated platelets using immunogold technique. CD41a antigen and factor XIII A subunit (FXIII‐A) are represented by 10 nm and 15 nm gold particles, respectively. A few 15 nm gold particles are depicted by small red arrows; for comparison a few 10 nm gold particles are indicated by small black arrows. A, Non‐stimulated whole platelet, (B) higher magnification of the selected area shown in (A), (C) a CVX+Thr stimulated whole platelet, (D) higher magnification of the selected area shown in (C). E–H, Microparticles formed from CVX+Thr activated platelets, (E, F) microparticles labeled for CD41a and cFXIII demonstrate at least partially intact membrane, (G, H) cFXIII positive microvesicles without CD41a membrane labeling. The images are representative of experiments performed with platelets from three different donors

We next studied FXIII‐A localization using high resolution immune electron microscopy (IEM). Figure 4A,B clearly demonstrate that non‐activated (resting) platelets fail to expose FXIII‐A to their surface, while they are intensively labeled for the surface marker CD41a. Activation of platelets with CVX+Thr reveals translocation of FXIII‐A from the cytoplasm to the external leaflet of the stimulated membrane (Figure 4C,D). Like on the immunofluorescent image, the accumulation of FXIII‐A within the cap‐like structure is observed by IEM, as well (Figure 4D). Microparticles of various size from 200 to 800 nm labeled positively for FXIII‐A (Figure 4E–H). The larger microparticles still retained their membrane structure, as evidenced by CD41a labeling, but also co‐stained for FXIII‐A (Figure 4E,F). Interestingly, microparticles of smaller size, which may represent extracellular cytoplasmic fragments, lack discernable membrane staining for CD41a, but they are heavily labeled for FXIII‐A (Figure 4G,H).

### 3.3 Non‐receptor mediated activation fails to transpose cellular FXIII to the surface of activated platelets and platelet microparticles

**FIGURE 5:**
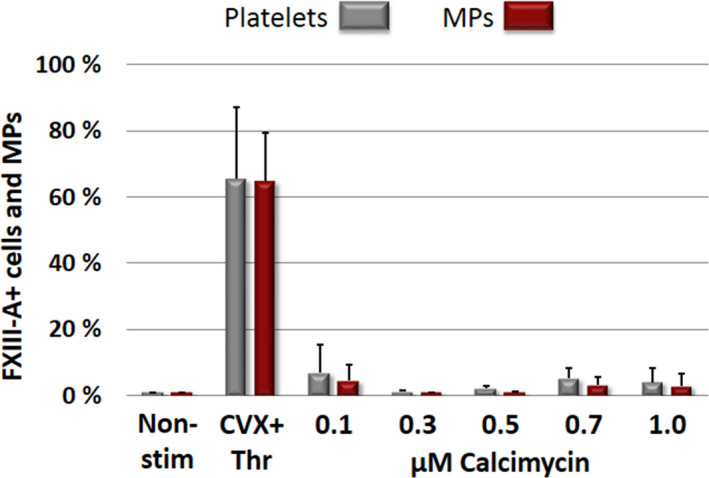
The lack of factor XIII A subunit (FXIII‐A) translocation to the surface of platelets and platelet microparticles by non‐receptor mediated activation. Non‐receptor mediated platelet activation was induced by various concentrations of calcimycin for 15 min. For comparison the effect of receptor mediated activation (CVX+Thr) is also shown. MPs, microparticles; Non‐stim, non‐stimulated platelets; CVX, convulxin; Thr, thrombin. The columns and error bars represent means ± standard deviations each calculated from the results of five experiments

The flow cytometric data presented in Figure 5 demonstrate that platelet activation by the Ca^2+^‐ionophore, calcimycin, that is, by directly elevating the intracellular free Ca^2+^ concentration, fails to switch on mechanisms responsible for the translocation of cFXIII to the surface of activated platelets and the formed microparticles during 15 min activation time. Figure S2 in supporting information demonstrates that extending the activation period did not change the situation considerably. Even after 45 min activation time only 6.6% ± 3.0% of platelets and 5.5% ± 2.2% of microparticles exposed cFXIII to the surface. For comparison, the considerably higher surface exposure of cFXIII induced by the combined stimulation of collagen and Thr receptors is also shown on the figure.

**FIGURE 6:**
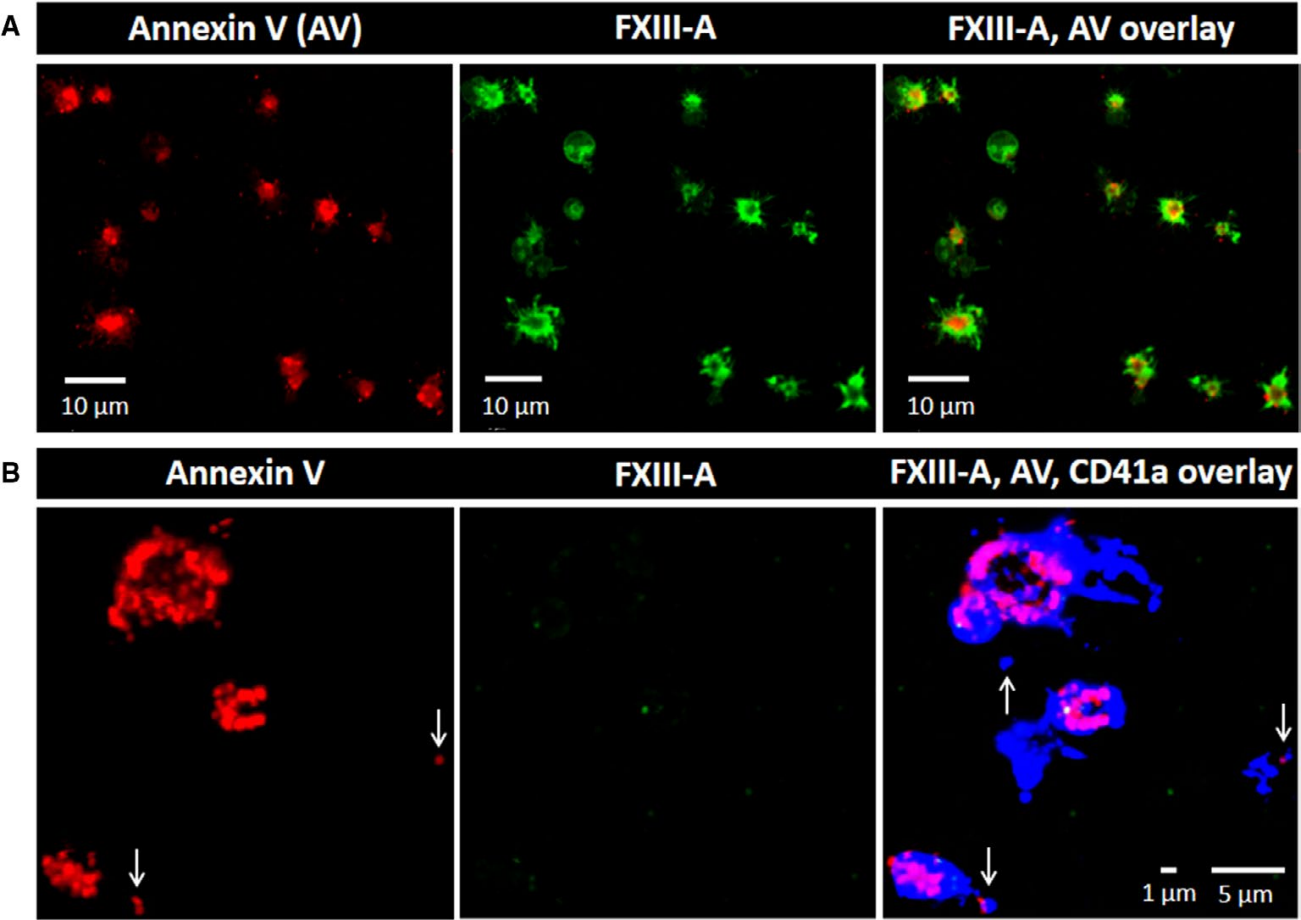
Labeling of permeabilized and non‐permeabilized platelets activated by Ca^2+^‐ ionophore. Platelets were stimulated with 0.7 µM calcimycin for 15 min. A, Platelets were permeabilized and labeled for phosphatidylserine (annexin V binding; red) and factor XIII A subunit (FXIII‐A; green). B, Non‐permeabilized platelets were stained for annexin V binding, FXIII‐A and CD41a (blue). Vertical arrows indicate microparticles. Note the absence of FXIII‐A staining on non‐permeabilized platelets as opposed to permeabilized ones. The images are representative of experiments performed with platelets from three different donors

Platelets undergoing non‐receptor mediated activation using Ca^2+^‐ionophore revealed staining for PS and FXIII‐A after permeabilization (Figure 6A) but FXIII‐A was located in the cytoplasm of these platelets. In contrast, non‐permeabilized platelets do not stain for FXIII‐A despite an intensive strong staining for PS and CD41a (Figure 6B). Similarly, no FXIII‐A positive microparticles were detected by immunofluorescent staining following calcimycin‐induced platelet activation. These data suggest that in the absence of receptor‐mediated stimulation FXIII‐A is hardly translocated to the outer membrane layer of the platelet membrane.

**FIGURE 7:**
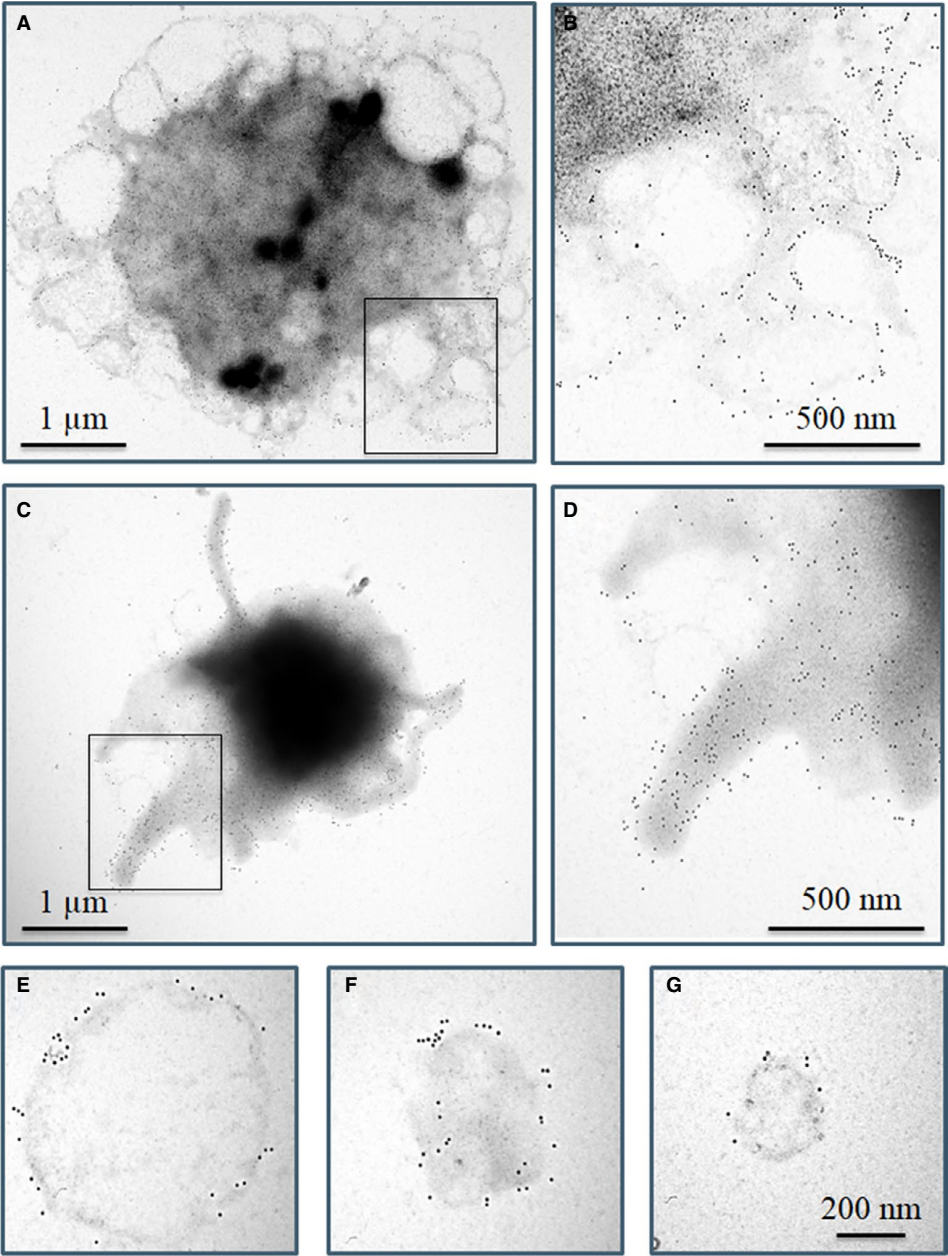
Surface labeling of Ca^2+^‐ionophore activated platelets using immunogold technique. Platelets were stimulated with 0.7 µM calcimycin. A‐D, Two different cellular structures of calcimycin activated platelets were observed, both of which were labeled for CD41a (10 nm gold particles). A,C, Whole platelets, (B,D) higher magnification of selected areas from (A,C). The absence of 15 nm gold particles on either platelet morphology indicates the lack of labeling for factor XIII A subunit (FXIII‐A). E–G, Microparticles of various size intensively labeled for CD41a failed to label for FXIII‐A. The images are representative of experiments performed with platelets from three different donors

IEM examination revealed two types of calcimycin‐activated platelets (Figure 7). Figure 7A,B show a platelet in the process of vesiculation at two magnifications, while Figure 7C,D demonstrate a platelet with intense pseudopod formation. Strong surface labeling for CD41a was present on both types of activated platelets but no surface exposure of FXIII‐A was evident. Microparticles of different size formed as the result of platelet activation by calcimycin (Figure 7E‐G). In this case the membrane and its labeling for CD41a remained intact, and no surface labeling for FXIII‐A could be detected. Note that activation by calcimycin failed to induce the formation of membrane‐free FXIII‐A positive microparticles/extracellular cytoplasmic fragments observed when platelets were activated by CVX+Thr.

### 3.4 Changes of cytosolic free Ca^2+^ concentration following platelet activation

**FIGURE 8:**
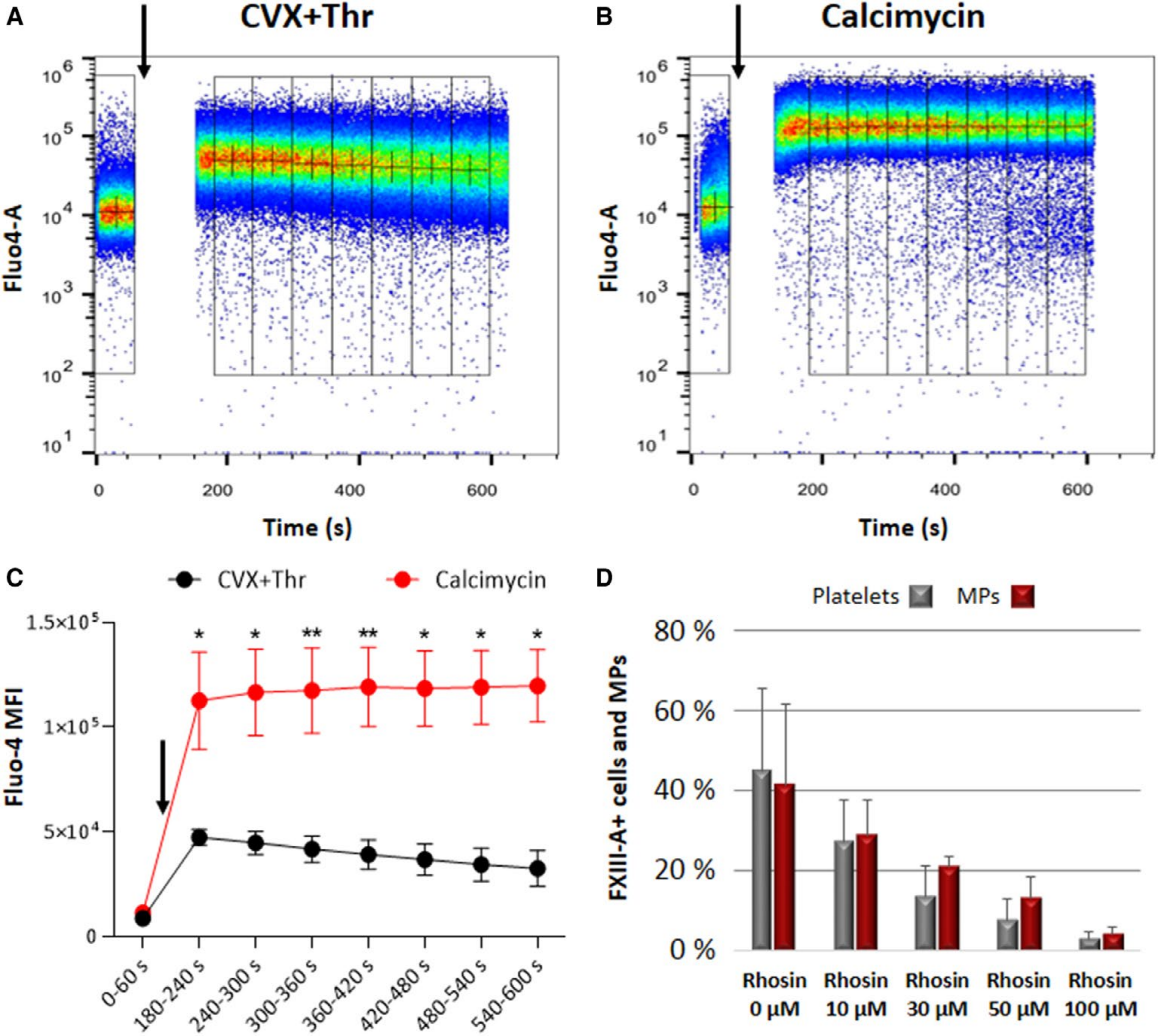
Agonists induced intracellular Ca^2+^ signal and the effect RhoA inhibitor on cFXIII transposition. Intracellular Ca^2+^ signal was measured by Fluo‐4 fluorescence in a flow cytometer. Representative pseudocolor plots of platelets treated either with (A) 125 ng/ml convulxin (CVX) plus 0.5 U/ml thrombin (Thr) or (B) 0.7 µM calcimycin. Administration of the agonists is indicated by arrows following a 60 s baseline acquisition. Fluorescence data were binned every 60 s (indicated by adjacent boxes), + signs represent the median of each bin. The time‐gap in the record corresponds to the addition of the agonists and the instrumental lag in data acquisition. C, Plots of median fluorescence intensity (MFI) for eight adjacent 60‐s‐long bins for platelets treated with CVX+Thr (in black), and the same dataset for calcimycin treatment (in red). Asterisks indicate significant change in the fractional fluorescence increase upon calcimycin versus CVX+Thr treatment (*P < .05, **P < .01). D, The effect of Rhosin (RhoA inhibitor) on the CVX+Thr induced surface translocation of cFXIII. Data shown in (C,D) represent the means of three individual measurements ± standard deviation

The effect of CVX+Thr or calcimycin treatment on the cytosolic free Ca^2+^ concentration of platelets was analyzed by flow cytometry (Figure 8A–C). Platelets were loaded with the Ca^2+^ indicator Fluo‐4‐AM and were labeled with fluorescence‐conjugated anti‐CD41a antibody. The latter signal was used for the gating of the flow cytometric signal for platelets (Figure S3). The first 60 s on panels A and B in Figure 8 shows the distribution of the Fluo‐4 fluorescence signal in resting platelets in the HEPES‐Ca^2+^ buffer. Data acquisition was then briefly interrupted for the application of the agonists (arrows, CVX+Thr, or calcimycin) and then resumed. The mean fluorescence of the Fluo‐4 signal showed an about 5‐fold increase upon the addition of the agonist cocktail CVX+Thr, which corresponds to an increase in the cytosolic free calcium concentration. Figure 8A also shows that the response of the cells to CVX+Thr was homogenous—most of the cells showed an immediate and marked increase in the Ca^2+^ concentration. The quantitative analysis of the median fluorescence intensity (MFI) for the 60‐s‐long bins shows that the increase in the cytosolic Ca^2+^ concentration was transient; the decline in the MFI as a function of time indicates this phenomenon. The same data acquisition protocol was applied when calcimycin was used as activating agent (Figure 8B). The increase in the Ca^2+^ concentration upon calcimycin addition was immediate and homogenous among platelets. The increase in the Ca^2+^ concentration was sustained over the data acquisition duration of 600 s, and the magnitude of the fluorescence increase was significantly greater than the one induced by CVX+Thr (Figure 8C).

### 3.5 The effect of RhoA and transglutaminase inhibitor on CVX+Thr induced cFXIII translocation

As calcimycin failed to induce cFXIII translocation, it was presumed that Ca^2+^ independent mechanisms are important in the surface exposure of cFXIII by CVX+Thr induced platelet activation. The small GTPase, RhoA, plays an important role in the Ca^2+^ independent part of G protein‐coupled receptor (GPCR) signaling and integrin‐mediated signaling.^24^, ^25^ For this reason, we investigated the effect of RhoA inhibitor, Rhosin, on cFXIII translocation (Figure 8D). The concentration‐dependent inhibition of cFXIII translocation by Rhosin underlines the importance of RhoA mediated Ca^2+^ independent pathways in the surface exposure of cFXIII during platelet activation induced through collagen and PAR receptors.

A further point to be addressed was if the transformation of cFXIII into an active transglutaminase (FXIIIa) was required for its transposition across the platelet membrane. Platelet activation by thrombin has been shown to result in the non‐proteolytic activation of part of cFXIII present in the intracellular compartment.^15^, ^26^ T101 is a powerful inhibitor of transglutaminase 2 and FXIIIa with an IC_50_ of about 0.25 μM. However, it cannot penetrate the membrane and is able to Inhibit only the active cFXIII fraction that was translocated to the outer membrane layer. We studied the effect of T101 on cFXIII externalization. The concentration dependent partial inhibition of cFXIII exposure by T101 suggests that the active form of cFXIII increases the translocation efficiency (Table S1 in supporting information). However, based on the data presented in Table S1 one can hardly decide if the decreased translocation is related to the inhibition of surface‐exposed cFXIIIa.

## 4 DISCUSSION

Platelets stimulated by receptor mediated activation using CVX+Thr and non‐receptor mediated activation with Ca^2+^‐ionophore share some common features including externalization of PS to the outer leaflet of the membrane and degranulation, observed as surface expression of the α‐granule protein P‐selectin. However, the morphology of platelets activated by the receptor mediated pathways and non‐receptor mediated mechanism were profoundly different. Following CVX+Thr stimulus approximately two‐thirds of the platelets assumed a balloon‐like morphology with a single cap protruding from the platelet body.^17^, ^27^, ^28^ In contrast, platelets stimulated with Ca^2+^‐ionophore formed vacuolized or pseudopod‐based structures. Similarly, the localization of cFXIII in platelets activated by receptor mediated and non‐receptor mediated stimuli is also different. In the absence of receptor stimulation cFXIII did not translocate to the surface of activated platelets and was not exposed on platelet microparticles. In theory, such a major difference might be due to a different extent of intracellular Ca^2+^ release induced through the receptor mediated and non‐receptor mediated activation pathways. However, the elevation of intracellular Ca^2+^ concentration due to calcimycin induced activation was even higher than the one induced by CVX+Thr. The results suggest it is not the elevation of Ca^2+^ in the cytosol that directs cFXIII to the outer membrane surface but rather the complex intracellular signaling mechanisms that accompany receptor stimulation. It is to be noted that Mattheij et al.,^18^ using another Ca^2+^‐ionophore, ionomycin, observed surface exposure of transglutaminase activity by a limited number of platelets. However, the high ionomycin concentration in their experiments, more than 10‐fold higher than that of the calcimycin in ours, and their long time of platelet stimulation might have induced less specific increased permeability of the platelet membrane.

A robust response resulting in the translocation of cFXIII requires coinciding signalization through collagen receptor and PARs.^25^ Both signalization pathways involve a sequence of Ca^2+^‐independent biochemical steps/mechanisms. Activation through the collagen receptor, GPVI, occurs via phosphotyrosine signaling cascade,^29^ which eventually leads to diacyl glycerol (DAG) production that activates Ser/Thr kinases of the protein kinase C family. At the cytoplasmic site PARs are bound to heterotrimeric G proteins, G_q_ and G_12/13_.^30^ Gα_q_ is involved in the activation of PLCβ and consequently in inositol triphosphate and DAG production, while Gα_12/13_ exerts its effect through RhoA activation. The inhibition of cFXIII translocation by the RhoA inhibitor, Rhosin, demonstrated its connection with a Ca^2+^‐independent part of signalization pathway.

PS translocated to the platelet surface shows circumferential location on the balloon‐like part of activated platelets and there is an abundance of PS concentrated in the cap.^28^ Platelets from FXIII‐A deficient patients are capable of formation of balloon‐shaped platelets with associated “cap,”^27^, ^31^ indicating that cFXIII does not participate in these processes. Clotting factors responsible for thrombin generation, FIXa, FX/FXa, FVIII, FVa, prothrombin plus fibrinogen, and other adhesive proteins are associated with the negatively charged phospholipid surface primarily due to PS translocated to the outer membrane layer in such “coated” platelets.^32^ On such a procoagulant surface transformation of adhered FX and prothrombin into active clotting factors becomes highly accelerated. Most clotting factors involved in thrombin generation come from extracellular sources, while fibrinogen and part of FV are also released from α‐granules. As a cytoplasmic protein cFXIII is not secreted through the classical secretory pathway or by degranulation of platelets.^33^ Still, its fate is reminiscent of the surface retention of alpha‐granular FV.^23^

Mitchel et al. demonstrated by flow cytometric and immunofluorescent methods that on CVX+Thr activated platelets FXIII‐A becomes surface exposed and in procoagulant platelets it is associated to the cap‐like structure.^17^, ^34^ Here we confirmed these findings and used high‐resolution IEM to visualize FXIII‐A in the cap. Interestingly, most recently it was shown that monocytes stimulated by interleukin 4 and 10 also transpose cFXIII to the membrane.^35^ Cordell et al. reported that in monocyte‐derived macrophages cFXIII becomes associated with Golgi proteins, which has been implicated in the delivery of non‐classically secreted proteins to the plasma membrane.^36^ The latter pathway might be involved in the appearance of FXIII‐A on the outer surface of activated platelets; however, further investigations are needed to explore the detailed mechanism of the trans‐bilayer movement of cFXIII during receptor mediated platelet activation. A further question is how surface‐exposed cFXIII becomes concentrated on the cap‐like structure protruding from activated platelets. FXIII zymogen present in the plasma might bind to activated platelet;^37^, ^38^ however, in our experimental set‐up no extracellular FXIII was present.

Platelets activated by Thr/TRAP or collagen/CVX and particularly by strong dual‐agonist stimulation release a wide range of extracellular vesicles that differ in size and morphology.^39^, ^40^ An excellent review on the formation, biochemical composition, and function of extracellular vesicles has been published by Gasecka et al. and they proposed terminology for microvesicle subspecies.^41^ Here, we investigated microvesicles in the range of 100–1000 nm for which the common terminology, microparticles, is used. cFXIII was documented in platelet microparticles some time ago;^42^ however, its surface exposure has not been investigated before. Our data has unequivocally demonstrated by flow cytometry, confocal immunofluorescence microscopy, and IEM that the majority of microparticles, formed as the result of platelet activation by CVX+Thr, expose cFXIII to their surface. It was also shown that FXIII‐A positive microparticles may aggregate and appear as circular or other irregularly clumped structures. Detailed high‐resolution IEM images revealed two types of FXIII‐A labeled microparticles. The larger ones of 400–800 nm diameter retained CD41a positivity, which suggests an intact membrane layer. The smaller ones, up‐to 200 nm, were CD41a negative but intensively labeled for FXIII‐A, suggesting that they are of cytoplasmic origin. Neither in non‐stimulated platelet preparations nor in calcimycin activated platelet preparations could such microparticles be detected. These results suggest that they derived from platelets undergoing robust receptor mediated activation.

The cross‐linking of α_2_‐antiplasmin and α_2_‐antiplasmin derived peptide to fibrin by cFXIII exposed to the surface of CVX+Thr activated platelets strongly indicates that this transglutaminase is in active form.^17^, ^18^ In an earlier study the transglutaminase induced binding of serotonin to several substrate proteins also indicated the presence of active FXIII on the platelet membrane.^43^ FXIII has been shown to become activated non‐proteolytically in the intracellular compartment during stimulation of platelets by thrombin.^15^, ^26^ It is likely that this robust stimulation simultaneously translocated the activated form of cFXIII to the outer cell surface as a result of intracellular signaling. It is feasible that non‐activated FXIII becomes exposed to the platelet surface and is subsequently proteolytically activated by thrombin when bound to the exterior of the platelet. However, without addition of exogenous thrombin, using TRAP‐6 and collagen as stimuli similar transglutaminase activity was noted.^17^ Collectively, these data suggest that at least part of cFXIII is translocated to the surface of stimulated platelets in activated form. These events localize cFXIIIa in a region of the thrombus where the transglutaminase can promote cross‐linking reactions to stabilize the forming platelet–fibrin aggregate.^17^ A further question was if activation of cFXIII is required for its transposition. The decreased surface availability of cFXIII in the presence of a transglutaminase inhibitor suggests some role of the active form.

Although no direct evidence was provided, it is likely that FXIII exposed on microparticles also represent activated cFXIII and play a role in intra‐thrombi fibrin stabilization. The small cytoplasmic fragments without surrounding membrane and intensively labeled for FXIII‐A are of particular interest. Their formation and exact structure are still to be explored and further studies on their involvement in thrombus stabilization might reveal an intriguing new mechanism.

This article has delineated a role for receptor mediated activation in the translocation of cFXIII to the outer membrane layer of activated platelet and this process is not linked to the exposure of procoagulant PS. It has been shown for the first time that cFXIII is present on the surface of microparticles. Despite the elevated intracellular Ca^2+^ levels, the Ca^2+^‐ionophore, calcimycin, failed to induce cFXIII translocation. This finding reveals the importance of Ca^2+^‐independent signaling mechanism(s) in the transposition of this cytoplasmic protein to the membrane surface. The decreased surface availability of cFXIII in CVX+Thr activated platelets pretreated by the RhoA inhibitor, Rhosin, also supports the importance of Ca^2+^‐independent mechanisms. Clearly further work is required to delineate the signaling pathways involved in the surface exposure of cFXIII and importantly to determine the contribution of cFXIII‐bearing microparticles in modulating thrombus stability.

## CONFLICTS OF INTEREST

The authors declare no competing financial interests.

## AUTHOR CONTRIBUTIONS

L.S. performed experiments, analyzed data, prepared figures, and was involved in writing the manuscript; I.B.D performed flow cytometric experiments; G.K. performed immune electron microscopic studies; M.C. carried out measurements of intracellular Ca^2+^ concentrations; J.K. supervised flow cytometric studies and critically evaluated the manuscript; M.A. performed and evaluated immune electron microscopic studies; G.P. designed and evaluated intracellular Ca^2+^ concentration measurements; H.B. performed and evaluated immunofluorescence experiments; N.J.M. was involved in data analysis, in critical evaluation, and revising the manuscript; L.M. designed the study, supervised the results, analyzed data, and wrote the manuscript.

## Supporting information

Supplementary Material
