# Supplementary material for: Activation mechanism dependent surface exposure of cellular factor XIII on activated platelets and platelet microparticles
Source: J Thromb Haemost. 2022 Feb 21;20(5):1223–35. doi: 10.1111/jth.15668 (PMC9303193; doi:10.1111/jth.15668)
Supplement: Supplementary file 1 — Supplementary Material [file JTH-20-1223-s001.pdf]

Supplementary material

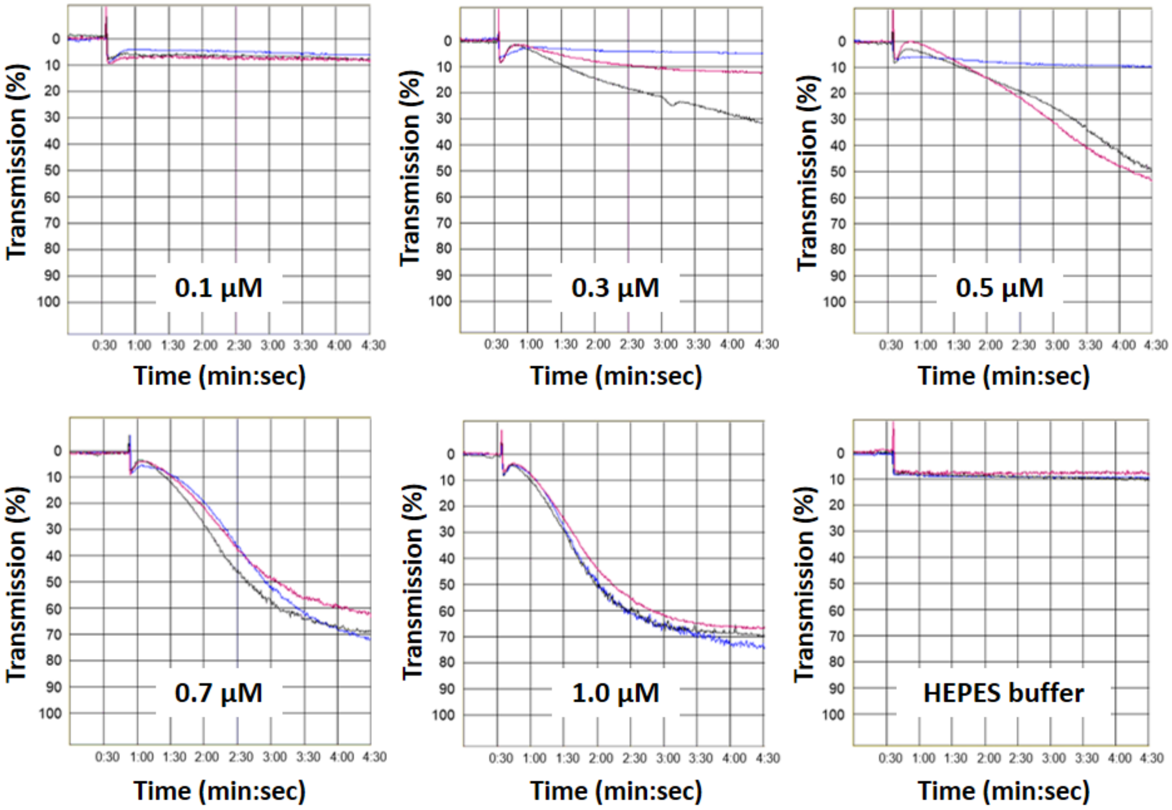

Figure S1. Ca^{2+}-ionophore (calcimycin) induced aggregation of gel-filtered platelets. The aggregation curves shown in three different colors represent platelets obtained from different individuals. Calcimycin concentrations are shown in bold.

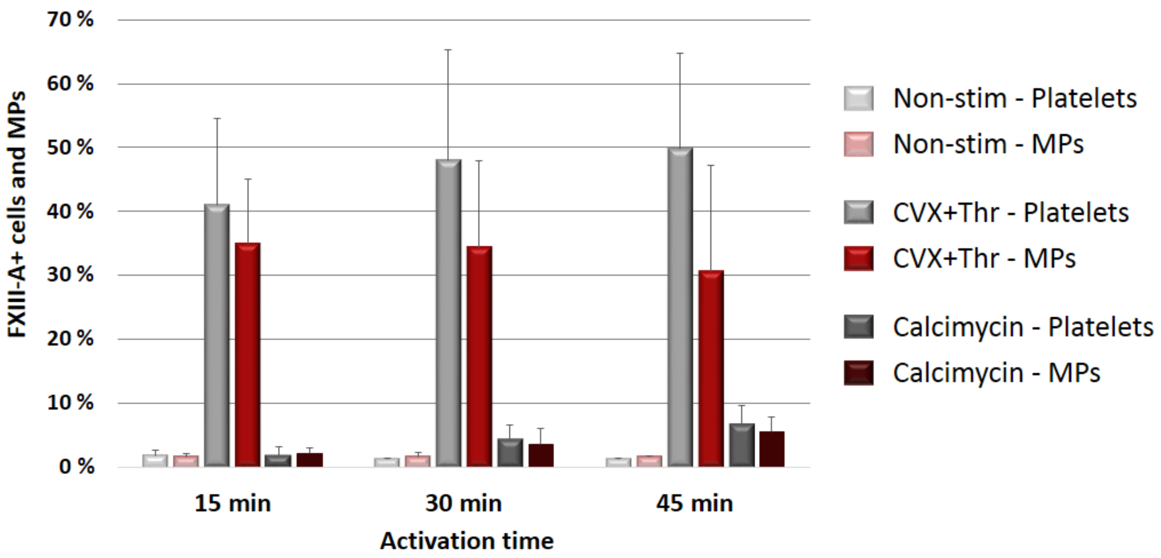

Figure S2. cFXIII tanslocation in platelets activated by CVX+Thr or calcimycin for various time. Non-stim: non-stimulated; MPs: microparticles; CVX: convulxin; Thr: thrombin

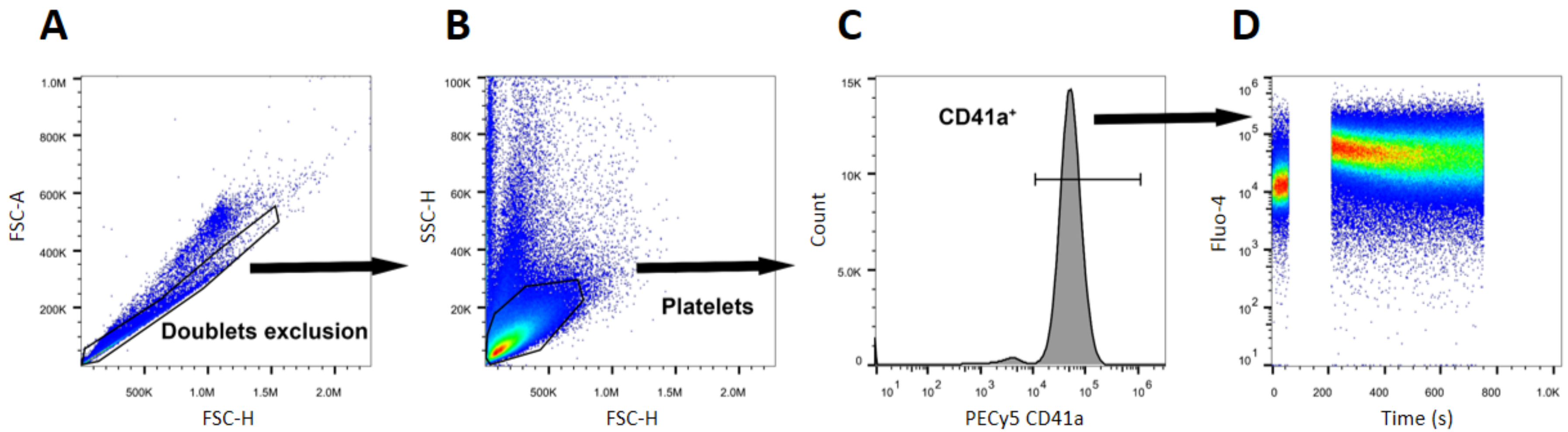

Figure S3. Gating strategy for human platelets. After doublets exclusion (FSC-H vs. FSC-A, panel A) and morphological gating (FSC-H vs. SSC-H, panel B), platelets were gated (panel C) and defined as CD41a+ cells. The CD41a+ gate was set based on the negative control (unstained cells, not shown). The Fluo-4 signal over time (D) is restricted to platelets gated in panel C .

% of platelets and microparticles with cFXIII exposed to the surface		
**T101 concentrations**	**Platelets Mean±SD**	**Microparticles Mean±SD**
0 μM	49±18	54±6
10 μM	30±16	33±14
20 μM	25±13	28±16
50 μM	26±18	26±16
100 μM	17±15	19±14
200 μM	16±13	20±15

Table S1. The effect of transglutaminase inhibitor, T101 on the translocation of cFXIII to the outer membrane layer in platelets activated by CVX+Thr.
